# The Novel PKC Activator 10-Methyl-Aplog-1 Combined with JQ1 Induced Strong and Synergistic HIV Reactivation with Tolerable Global T Cell Activation

**DOI:** 10.3390/v13102037

**Published:** 2021-10-09

**Authors:** Ayaka Washizaki, Megumi Murata, Yohei Seki, Masayuki Kikumori, Yinpui Tang, Weikeat Tan, Nadita P. Wardani, Kazuhiro Irie, Hirofumi Akari

**Affiliations:** 1 Primate Research Institute, Kyoto University, Inuyama 484-8506, Japan; washizaki.ayaka.4r@kyoto-u.ac.jp (A.W.); murata.megumi.4s@kyoto-u.ac.jp (M.M.); yseki@niid.go.jp (Y.S.); lorenpuipui@gmail.com (Y.T.); tan.keat.64x@st.kyoto-u.ac.jp (W.T.); nadita.wardani@alumni.i3l.ac.id (N.P.W.); 2 Graduate School of Agriculture, Kyoto University, Kyoto 606-8502, Japan; k9morin@gmail.com (M.K.); irie.kazuhiro.2z@kyoto-u.ac.jp (K.I.); 3 Department of Biomedicine, Indonesia International Institute for Life Sciences, Jakarta 13210, Indonesia; 4 Institute for Frontier Life and Medical Sciences, Kyoto University, Kyoto 606-8507, Japan

**Keywords:** HIV, shock and kill, latency-reversing agents, PKC activator, 10-methyl-aplog-1

## Abstract

The presence of latent human immunodeficiency virus (HIV) reservoirs is a major obstacle to a cure. The “shock and kill” therapy is based on the concept that latent reservoirs in HIV carriers with antiretroviral therapy are reactivated by latency-reversing agents (LRAs), followed by elimination due to HIV-associated cell death or killing by virus-specific cytotoxic T lymphocytes. Protein kinase C (PKC) activators are considered robust LRAs as they efficiently reactivate latently infected HIV. However, various adverse events hamper the intervention trial of PKC activators as LRAs. We found in this study that a novel PKC activator, 10-Methyl-aplog-1 (10MA-1), combined with an inhibitor of bromodomain and extra-terminal domain motifs, JQ1, strongly and synergistically reactivated latently infected HIV. Notably, higher concentrations of 10MA-1 alone induced the predominant side effect, i.e., global T cell activation as defined by CD25 expression and pro-inflammatory cytokine production in primary CD4+ T lymphocytes; however, JQ1 efficiently suppressed the 10MA-1-induced side effect in a dose-dependent manner. Considering the reasonable accessibility and availability of 10MA-1 since the chemical synthesis of 10MA-1 requires fewer processes than that of bryostatin 1 or prostratin, our results suggest that the combination of 10MA-1 with JQ1 may be a promising pair of LRAs for the clinical application of the “shock and kill” therapy.

## 1. Introduction

Antiretroviral therapy (ART) development turns human immunodeficiency virus (HIV) infection to a manageable chronic disease. In fact, ART may successfully control plasma viremia to undetectable levels. However, the latent reservoirs that harbor replication-competent HIV remain in infected individuals under ART, representing a significant obstacle to HIV cure. The “shock and kill” therapy is based on the concept that latency-reversing agents (LRAs) reactivate the latently infected HIV of ART controllers, followed by elimination due to HIV-associated cell death or killing by virus-specific cytotoxic T lymphocytes [1,2,3]. Thus far, various LRAs have been reported, which can be classified based on their mechanisms of action [4].

Abundant histone deacetylation and methylation occur in the proviral DNA of latently HIV-infected quiescent CD4+ T cells. This induces heterochromatic structure [5]. Histone deacetylase inhibitors (HDACi), such as romidepsin and vorinostat, block histone deacetylase, leading to histone acetylation [5,6]. Inhibitors of bromodomain and extra-terminal domain motifs (BET), such as JQ1, I-BET, and OTX015, also induce epigenetic modification leading to HIV reactivation. BET inhibitors block bromodomain-containing protein 4 (BRD4) from recruiting positive transcription elongation factor b (P-TEFb) to acetylated histones, which promotes binding of Tat protein to P-TEFb, and their recruitment to the trans-activation response (TAR) element of HIV long terminal repeat (LTR) [4,7,8,9,10]. Alternatively, NF-κB activation and translocation into the nucleus lead to HIV reactivation in the latency [9,11]. LRAs, such as the second mitochondria-derived activator of caspase mimetics, Toll-like receptor (TLR) 1/2 agonists, and especially protein kinase C (PKC) activators, such as bryostatin 1, prostratin, and ingenol-3-angelate, efficiently reactivate HIV via the NF-κB-dependent pathway [12,13,14,15,16]. PKC activation induces the phosphorylation of IκB, leading to NF-κB activation and translocation of activated NF-κB into the nucleus [17,18]. Spina et al. showed that PKC activators reactivated latently infected HIV through the NF-κB pathway from all latency models tested, whereas other classes of LRA did not. Therefore, PKC activators are considered robust LRAs [11,19,20].

However, it was reported that PKC activators developed adverse events, such as fever or headache, in clinical trials as anticancer agents [21,22,23,24]. Assuming that healthy ART-treated individuals living with HIV would be the candidates for shock and kill therapy, the efficacy versus safety of PKC activators should be carefully considered [25]. Because of their risk, the efficacy of PKC activators on HIV reactivation has been scarcely examined as an intervention trial [21], which is in contrast with those of HDACi and TLR agonists [6,26,27].

A possible solution for overcoming this hurdle is using PKC activators combined with BET inhibitors. Combining a PKC activator and a BET inhibitor synergistically reactivated HIV [4,13,28] and reduced global T cell activation and pro-inflammatory cytokine expression [28,29,30]. These results suggest that the combined use of a PKC activator and a BET inhibitor may overcome the safety issue of PKC activators as LRAs. However, most previous studies did not dose-dependently examine their effects on synergistic HIV reactivation in parallel with global T cell activation. Thus, it remains to be elucidated whether a particular combination of PKC activators and BET inhibitors with their optimized concentrations could achieve efficient HIV reactivation together with lowered and tolerable side reactions.

10-Methyl-aplog-1 (10MA-1) is a simplified analog of aplysiatoxin (ATX), a naturally occurring PKC ligand with tumor-promoting activity, such as phorbol 12-myristate 13-acetate (PMA), and has a potent ability to activate PKC isozymes [31,32]. Although the binding potency of 10MA-1 against PKCdelta is similar to that of ATX, its adverse effects (pro-inflammatory and tumor-promoting activities) are circa 100 times weaker than those of ATX [31]. Notably, the chemical synthesis of 10MA-1 requires fewer processes than that of bryostatin 1 or prostratin, leading to reasonable accessibility and availability of 10MA-1 [33]. We therefore sought to address the following questions; (i) whether 10MA-1 combined with JQ1, a representative BET inhibitor, would efficiently reactivate HIV, and if so, (ii) whether certain optimized concentrations of 10MA-1 and JQ1 could successfully achieve efficient HIV reactivation in parallel with lowered and tolerable side reactions.

## 2. Materials and Methods

### 2.1. Cell Lines

Jurkat 1G5 is derived from a leukemic T cell line Jurkat with a luciferase gene driven by an HIV-1 LTR [34]. J-Lat 9.2 is also a derivative of Jurkat and has a single copy of latent HIV proviral genome with one copy of green fluorescent protein (GFP) gene at the *nef* region [35]. J-Lat 9.2 produces noninfectious HIV-1. Jurkat 1G5 and J-Lat 9.2 were obtained from the NIH AIDS Reagent Program (Manassas, VA, USA). M8166, a subclone of C8166 T cell line, has increased susceptibility to simian immunodeficiency virus [36]. These cell lines were cultured in R-10 composed of RPMI-1640 (Sigma Aldrich, Darmstadt, Germany) medium supplemented with 10% fetal bovine serum, 292 ng/mL L-glutamine, 100 U/mL penicillin G, and 100 µg/mL streptomycin (FUJIFILM Wako Pure Chemical Corporation, Osaka, Japan) at 37 °C in 5% CO_2_ atmosphere.

### 2.2. Reagents

10MA-1 is an analog of ATX classified into a PKC activator isolated from sea hare (*Stylocheilus longicauda*) [37]. 10MA-1 was synthesized as described previously [33]. 12-Deoxyphorbol 13-phenylacetate (DPP), phorbol 12-myristate 13-acetate (PMA), bryostatin 1, and prostratin was purchased from Sigma-Aldrich (Darmstadt, Germany), and JQ1 were from Cayman Chemical (Ann Arbor, MI, USA). The suberoylanilide hydroxamic acid (SAHA) was provided by NIH AIDS Reagent Program.

### 2.3. Evaluation of HIV-1 Reactivation by LRAs

Jurkat 1G5 cells were cultured in 96-well plates at 5 × 10^4^ cells per well in 100 µL of R-10 media. The cells were treated with LRAs. After 24 h post-treatment, HIV-1 reactivation was measured by luciferase activity using the luciferase assay system (Promega, Madison, WI, USA) following the manufacturer’s instructions.

J-Lat 9.2 cells were cultured and treated with LRAs as described above. However, at 48 h post-treatment, the amount of p24 capsid protein in the culture supernatant was measured via HIV-1 p24 antigen ELISA v.2.0 (ZeptoMetrix Corporation, Buffalo, NY, USA) following the manufacturer’s instructions, and the cells were assessed for GFP expression by flow cytometry. The cells were washed with phosphate-buffered saline (PBS) supplemented with 1% FBS and resuspended in 1× BD CellFIX (BD Bioscience, San Jose, CA, USA). The percentages of GFP-positive cells were measured on BD FACSVerse (BD Bioscience, San Jose, CA, USA), and data were analyzed using FlowJo v.10.0 (FlowJo, Ashland, OR, USA).

### 2.4. Cytotoxicity Assay

M8166 or Jurkat was seeded into 96-well plates at 5 × 10^4^ cells per well and was treated with LRAs as described above. After 24 h of incubation, the viability of the cells was determined using the Cell Counting Kit-8 (Dojindo, Kumamoto, Japan), a highly water-soluble tetrazolium salt (WST-8)-based assay for quantifying viable cells. WST-8 (10 µL) solution was added to 100 µL of cell suspension. After 2 h of incubation, the absorbance at 450 nm was measured using a microplate reader BIO-RAD Model 680 (Bio-Rad, Hercules, CA, USA).

### 2.5. Statistical Analysis

We used the Bliss independence model to calculate synergy as previously reported [30]. The Bliss independence model is defined by the equation *f_axy_*, _P_ = *f_ax_* + *f_ay_* − (*f_ax_*)(*f_ay_*), where *f_axy_*_, P_ is the predicted fraction affected by combining drug *x* and drug *y* given the experimentally observed fraction affected for drug *x* (*f_ax_*) and drug *y* (*f_ay_*) individually. The experimentally observed fraction, affected by a combination of drug *x* and drug *y* (*f_axy_*_, O_), is compared with the predicted fraction is affected, which is computed using the Bliss model (*f_axy_*_, O_) as follows: Δ*f_axy_* = *f_axy_, _O_* − *f_axy_*_, P_. If Δ*f_axy_* < 0 with statistical significance. Then, the combined effect of the two drugs will exceed that predicted by the Bliss model, and the drug combination displays synergy. If Δ*f_axy_* = 0, then the drug combination follows the Bliss model for independent action. If Δ*f_axy_* > 0 has statistical significance, then the combined effect of the two drugs is less than that predicted by the Bliss model, and the drug combination displays antagonism.

To analyze the synergistic effect of LRAs on reactivation of HIV-1 from Jurkat 1G5, we calculated the percentage of relative luciferase unit (RLU) induced by LRA *x* or LRA *y* as the RLU shown by the combination of 3.2 µM JQ1 and 3.2 µM prostratin was 100%. Then, the percentages of RLU induced by LRA *x* or LRA *y* were defined as *fa_x_* or *fa_y_*, respectively, and those of LRA treated with a combination of LRA *x* and LRA *y* were defined as *fa_xy_*_,o_. Alternatively, to analyze the synergistic effect of LRAs on J-Lat 9.2, the percentages of GFP-positive J-Lat 9.2 treated with each LRA, LRA *x*, or LRA *y* were defined as *fa_x_* or *fa_y_*, respectively. In contrast, those of GFP-positive cells treated with a combination of LRA *x* and LRA *y* were defined as *fa_xy_*_,o_.

### 2.6. Preparation of Peripheral Blood Mononuclear Cells (PBMCs)

PBMCs were separated from the peripheral blood of three healthy cynomolgus macaques (*Macaca fascicularis*) with a standard Ficoll density gradient method. The animals were confirmed to be negative for B virus, simian T cell leukemia virus type 1, simian immunodeficiency virus, and simian retroviruses, following the protocols of experimental procedures approved by the Animal Welfare and Animal Care Committees of the Primate Research Institute of Kyoto University (approval number: 2020-148, 3 March 2020). Blood collection was performed under ketamine hydrochloride anesthesia with medetomidine, followed by atipamezole for quick recovery from anesthesia.

### 2.7. Evaluation of PBMCs Activation by LRAs

The PBMCs were seeded at 2 × 10^5^ cells per well into 96-well plates and treated with LRAs at the final concentration. After 24 h incubation at 37 °C, the cells and culture supernatants were harvested. The cells were washed with PBS supplemented with 1% FBS and were stained with a cocktail of mouse antihuman monoclonal antibodies; CD3-APC-Cy7, CD4-PerCP-Cy5.5, CD69-PE (BD Pharmingen, San Jose, CA, USA), CD8-FITC (Dako, Agilent, Santa Clara, CA, USA), and CD25-Pacific Blue (BioLegend, San Diego, CA, USA). The treated cells were assessed for fluorescence intensity by FACSVerse and analyzed by FlowJo v.10.0. Also, the culture supernatants were evaluated for the levels of TNF-α, IL-1β, IL-6, and IL-8 using ELISA kits (Invitrogen, Carlsbad, CA, USA) following the manufacturer’s instructions.

## 3. Results

### 3.1. The Effect of 10MA-1 on HIV Reactivation in HIV Latency Model Cell Lines

**Figure 1:**
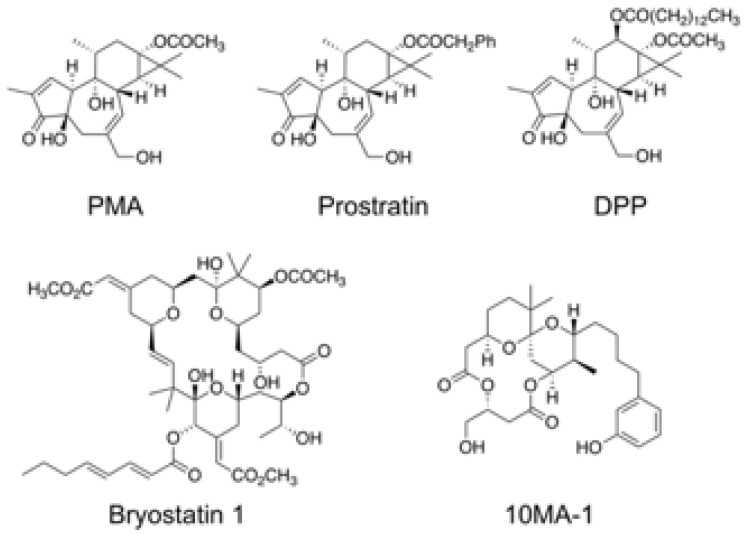
Chemical structure of 10MA-1 and related PKC activators. Chemical structures of PMA, prostratin, DPP, bryostatin 1, and 10MA-1 are shown.

**Figure 2:**
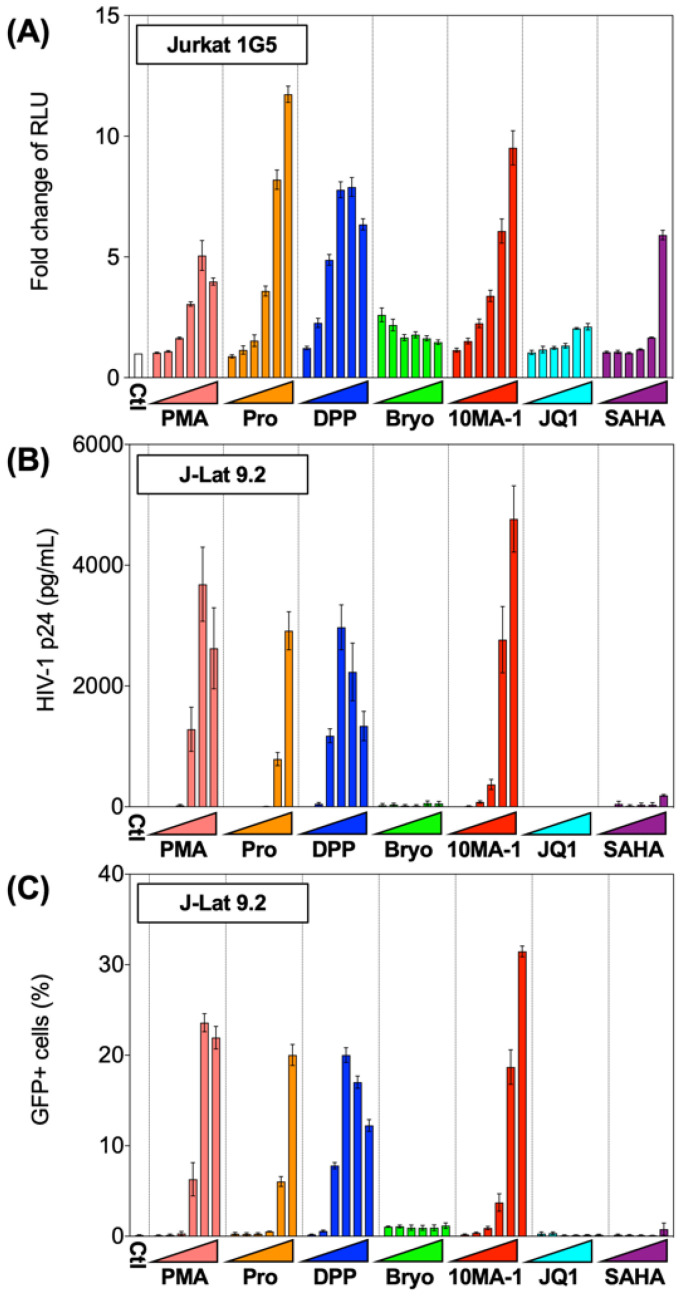
Reactivation of HIV by LRAs in Jurkat 1G5 and J-Lat 9.2. (**A**) Jurkat 1G5 was treated with LRAs, including prostratin (Pro), DPP, bryostatin 1 (Bryo), 10MA-1, JQ1, and SAHA. Fold changes of RLU between 1G5 treated with and without the LRAs for 24 h are shown. (**B**) J-Lat 9.2 was treated with the same LRAs as shown in (**A**) for 48 h, then (**B**) p24 amount in the culture supernatants and (**C**) GFP expression was measured. We performed experiments in triplicate, and averages and standard deviations of the results were indicated. The LRAs were used in these assays at the concentration of 10 nM, 32 nM, 100 nM, 320 nM, 1 µM, or 3.2 µM. PMA was used as a positive control at the concentration of 0.1 nM, 0.32 nM, 1 nM, 3.2 nM, 10 nM, or 32 nM.

The chemical structure of 10MA-1 and related PKC activators including PMA, prostratin, DPP, bryostatin 1, and 10MA-1 is shown in Figure 1. To investigate whether 10MA-1 would reactivate latently infected HIV, Jurkat 1G5 was treated with LRAs, including prostratin, DPP, bryostatin 1, 10MA-1, JQ1, and SAHA. 10MA-1 induced luciferase activity in a dose-dependent manner, and the fold increase in relative luciferase unit (RLU) induced by 10MA-1 (approximately 10-fold) was comparable to that of prostratin and DPP. Simultaneously, JQ1, bryostatin 1, and SAHA performed less efficiently (Figure 2A). When an alternative indicator cell line J-Lat 9.2 was treated with 10MA-1, both the p24 amount in the culture supernatants and frequency of GFP+ cells increased in a dose-dependent manner. The effect of 10MA-1 was stronger than that of other PKC activators (Figure 2B,C). The manner of HIV reactivation in the indicator cell lines varied according to PKC activators; bryostatin 1 did not show remarkable reactivation as previously reported (Figure 2) [28]. In this regard, when Jurkat 1G5 was treated with PKC activators, followed by measuring RLU at 6, 12, and 24 h post-treatment, bryostatin 1 showed a peak of RLU at 6 h post-treatment, and it dramatically dropped after that (Figure S1A). Alternatively, 10MA-1 induced RLU in Jurkat 1G5 by approximately 7-fold of the background level within 6 h, followed by a gradual increase until 24 h in a dose-dependent manner (Figure S1B). There were similar kinetics in prostratin and DPP (Figure S1C,D). These results indicated that the kinetics of HIV reactivation induced by bryostatin 1 was different from those by other PKC activators. We therefore employed prostratin as a reference PKC activator for further experiments due to the similarity in reactivation kinetics.

### 3.2. The Combined Treatment of 10MA-1 with JQ1 Synergistically Reactivated Latent HIV

**Figure 3:**
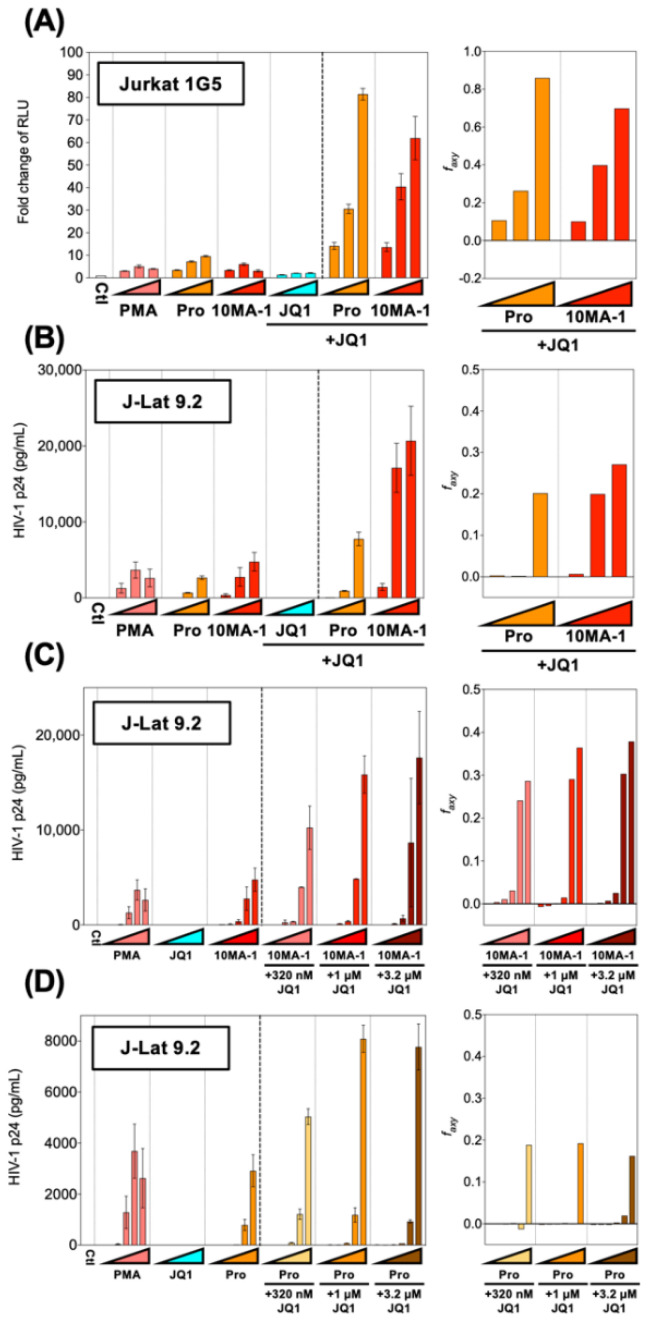
Combined treatment of 10MA-1 and JQ1 synergistically reactivated latent HIV. (**A**) Jurkat 1G5 was treated for 24 h with prostratin (Pro) or 10MA-1 with or without JQ1, as indicated in the figure. Fold changes of RLU in 1G5 and synergy of the combination treatment are shown. (**B**) J-Lat 9.2 was treated with the same LRAs as shown in (**A**) for 48 h, then p24 amount in the culture supernatants and synergy of the combined treatment was measured. We performed experiments in triplicate; averages and standard deviations of the results are shown. The LRAs were used in (**A**,**B**) at a concentration of 320 nM, 1 µM, or 3.2 µM for each LRA with or without 3.2 µM of JQ1. PMA was used as a positive control at a concentration of 3.2 nM, 10 nM, or 32 nM. (**C**,**D**) J-Lat 9.2 was treated with 10MA-1 (**C**) or prostratin (Pro) (**D**) with or without JQ1 for 48 h, then p24 amount in the culture supernatants and synergy of the combination treatment were measured. Experiments were performed in triplicate; averages and standard deviations of the results are shown. The LRAs were used in (**C**,**D**) at a concentration of 10 nM, 32 nM, 100 nM, 320 nM, 1 µM or 3.2 µM for each LRA or along with 320 nM, 1 µM, or 3.2 µM of JQ1, respectively. PMA was used as a positive control at a concentration of 0.1 nM, 0.32 nM, 1 nM, 3.2 nM, 10 nM, or 32 nM. The Bliss independence model statistically calculated synergies of the combined treatment. If Δ*fa_xy_* > 0, the combination displays synergy. Alternatively, if ∆*fa_xy_* < 0, the combination displays antagonism.

HIV reactivation by PKC activators was synergistically enhanced by combined treatment with BET inhibitors [4,13,28]. To see if HIV reactivation by 10MA-1 would be synergistically augmented by JQ1, popular BET inhibitors Jurkat 1G5 and J-Lat 9.2 were treated with 10MA-1 combined with JQ1. The treatment of 3.2 µM 10MA-1 and 3.2 µM JQ1 induced RLU approximately 60-fold of the background level. Similarly, the treatment of 3.2 µM prostratin and 3.2 µM JQ1 induced RLU up to 80-fold (Figure 3A). Similar results were observed in J-Lat 9.2 (Figure 3B).

Next, we investigated the effect of lower concentrations of JQ1 on HIV reactivation in J-Lat 9.2. The results indicated that 3.2 µM and 1 µM and 320 nM JQ1 synergistically reactivated HIV when 1 µM or 3.2 µM 10MA-1 was treated together (Figure 3C). Further, the combined treatment of 320 nM, 1 µM, or 3.2 µM JQ1 showed synergistic reactivation of HIV only when 3.2 µM prostratin was used (Figure 3D). Consistent results were obtained as shown above when the effect of the combined treatment was evaluated as percentages of GFP+ populations in J-Lat 9.2 cells (Figure S2). These results indicated that relatively lower concentrations of JQ1 were effective for synergistic HIV reactivation in a combined treatment with 10MA-1.

### 3.3. The Cytotoxicity of LRAs

**Figure 4:**
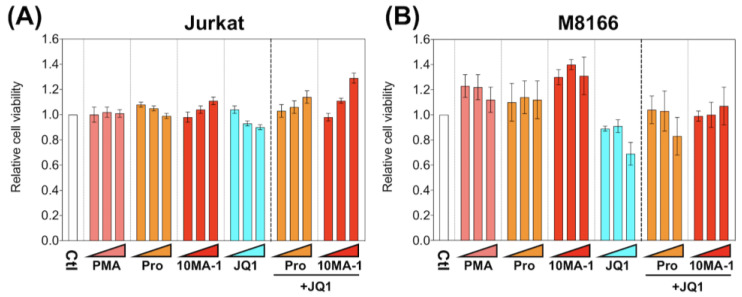
Cytotoxicity of LRAs in T cell lines. (**A**) Jurkat and (**B**) M8166 were treated for 24 h with each LRA or along with 3.2 µM of JQ1, then relative numbers of living cells were measured by Cell Counting Kit-8. We performed experiments in triplicate; averages and standard deviations of the results are shown. Moreover, the relative cell viability was calculated by dividing the average absorbance at 450 nm in the culture media of the treated cells by that of untreated cells. The LRAs were used in these assays at a concentration of 320 nM, 1 µM, or 3.2 µM, with or without 3.2 µM of JQ1. PMA was used as a positive control at a concentration of 3.2 nM, 10 nM, or 32 nM.

To investigate the cytotoxic effect of 10MA-1 compared with other LRAs, we examined the viability of M8166 and Jurkat 24 h after the treatment of LRAs (Figure 4). The treatment of 10MA-1 or in combination with JQ1 did not show cytotoxicity in either cell line. Similar results occurred for prostratin. Instead, the cytotoxicity by JQ1 was alleviated when the PKC activators were treated combined with JQ1. These results suggest that the nonspecific cytotoxicity caused by 10MA-1 alone or combined with 10MA-1 and JQ1 is tolerable.

### 3.4. The Combined Treatment of 10MA-1 with JQ1 Efficiently Suppressed Global T Cell Activation

**Figure 5:**
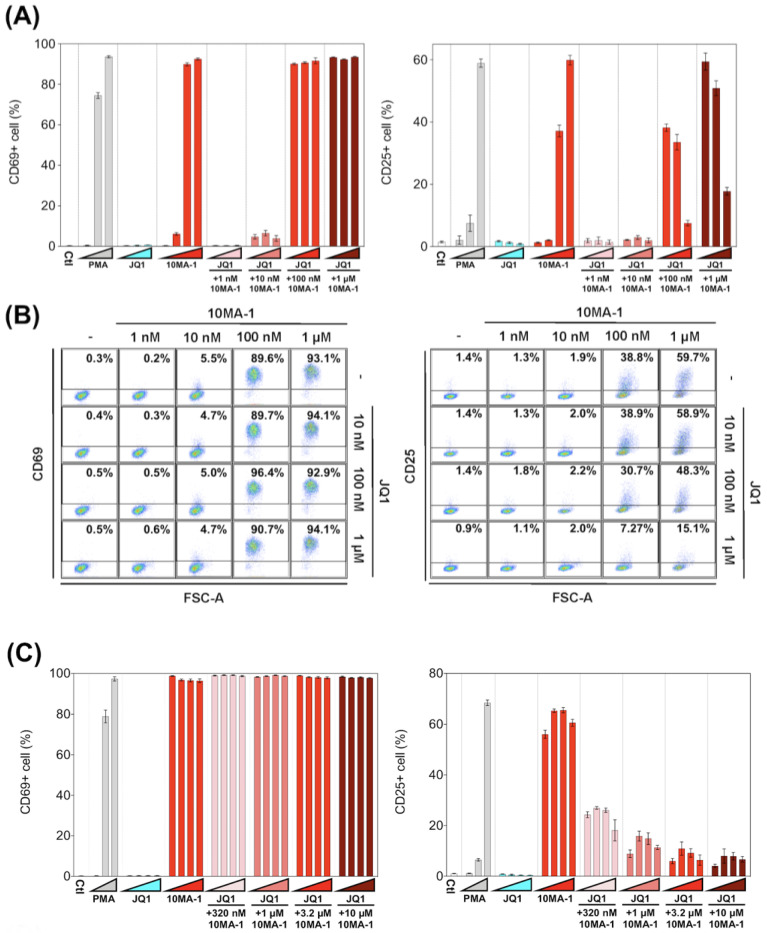

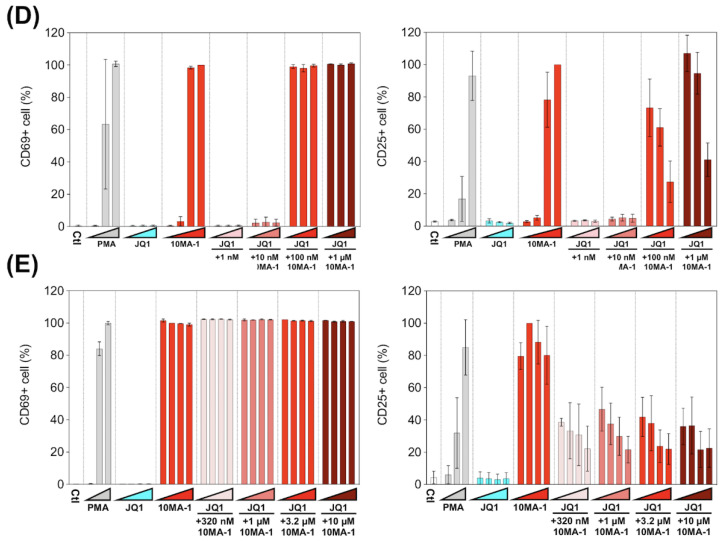
T cell activation induced by the combined treatment of LRAs. (**A**) PBMCs were untreated or treated with increasing concentrations of PMA (0.1 nM, 1 nM, or 10 nM), JQ1 (10 nM, 100 nM, or 1 µM), 10MA-1 (1 nM, 10 nM, 100 nM, or 1 µM), or JQ1 (10 nM, 100 nM, or 1 µM) and 10MA-1 (1 nM, 10 nM, 100 nM, or 1 µM) for 24 h. The CD4+ T lymphocytes were measured for the positivity of CD69 (left) and CD25 (right) by flow cytometry. We performed experiments in triplicate; the averages and standard deviations of the results obtained from the PBMCs of a macaque (MF-1) are shown. (**B**) Representative dot plot data by flow cytometry for (**A**) are shown. (**C**) PBMCs were untreated or treated with an increasing concentration of PMA (0.1 nM, 1 nM, or 10 nM), JQ1 (320 nM, 1 µM, 3.2 µM, or 10 µM), 10MA-1 (320 nM, 1 µM, 3.2 µM, or 10 µM), or JQ1 (320 nM, 1 µM, 3.2 µM, or 10 µM) and 10MA-1 (320 nM, 1 µM, 3.2 µM, or 10 µM) for 24 h. CD4+ T lymphocytes were examined for the positivity of CD69 (left) and CD25 (right) by flow cytometry. We performed experiments in triplicate; the averages and standard deviations of the results obtained from the PBMCs of a macaque (MF-1) are shown. (**D**) Averages of the results obtained from PBMCs of three monkeys regarding CD69 and CD25 expression in the CD4+ T lymphocytes treated along with LRAs. The averages of the data from PBMCs of three monkeys in each experimental setting employed in A and C are indicated as D and E, respectively. The data were calculated as relative percentages of CD69 or CD25-positive cells; the 1 µM or 10 µM 10MA-1 treatment data were set to 100%. (**E**) Averages of the results obtained from PBMCs of three monkeys regarding CD69 and CD25 expression in the CD4+ T lymphocytes treated along with LRAs. The averages of the data from PBMCs of three monkeys in each experimental setting employed in C are indicated. The data were calculated as relative percentages of CD69 or CD25-positive cells; the 1 µM or 10 µM 10MA-1 treatment data were set to 100%.

We examined the effect of 10MA-1 on T cell activation. PBMCs were treated with each LRA or combined with JQ1 for 24 h, followed by evaluation in the expression of an early activation marker, CD69, and a late (global) activation marker, CD25, in CD4+ T lymphocytes by flow cytometry. 10MA-1 induced CD69 expression in CD4+ T cells in a dose-dependent manner; the cells untreated or treated with 1 nM 10MA-1 were primarily negative for CD69, while 5.5%, 89.6%, and 93.1% of the cells expressed CD69 by the treatment of 10 nM, 100 nM, or 1 µM 10MA-1, respectively. Increasing concentrations of JQ1 had no influence on CD69 expression in the cells. The combined treatment with JQ1 did not influence the CD69 positivity of the cells treated with 10MA-1, irrespective of JQ1 concentrations (Figure 5A,B). In contrast to the results of CD69, the combination treatment of 10MA-1 with JQ1 reduced the frequency of the cells expressing CD25 in a JQ1 concentration-dependent manner. The results indicated that 38.8% of the cells treated with 100 nM 10MA-1 became positive for CD25. However, 30.7% and 7.3% of the cells treated with 100 nM 10MA-1 and 100 nM or 1 µM of JQ1 did so. Similarly, 59.7% of the cells treated with 1 µM 10MA-1 alone became positive for CD25, and 48.3% and 15.1% of the cells treated with 1 µM 10MA-1 with 100 nM or 1 µM JQ1 did so (Figure 5A,B). Consistent results were observed among PBMCs from three macaques (Figure 5D and Figure S3). These results suggest that global T cell activation, as shown by CD25 expression, was successfully suppressed through the combined treatment of 10MA-1 with JQ1. However, it was sufficient to trigger early T cell activation, as indicated by CD69 expression.

Next, we investigated whether the effect of 10MA-1 at concentrations more than 1 µM on the global T cell activation could be inhibited by the combination treatment of JQ1. PBMCs were treated for 24 h with 320 nM, 1 µM, 3.2 µM, or 10 µM 10MA-1 with or without 320 nM, 1 µM, 3.2 µM, or 10 µM JQ1, respectively, followed by evaluation of CD69 and CD25 expression in CD4+ T cells. The global T cell activation induced by 10MA-1 was efficiently inhibited by 320 nM or more concentrations of JQ1 in a dose-dependent manner. However, JQ1 treatment did not affect the early T cell activation induced by 10MA-1 (Figure 5C). Among PBMCs, there were consistent results from three macaques (Figure 5E and Figure S4).

### 3.5. Combined Treatment of 10MA-1 with JQ1 Efficiently Reduced Pro-Inflammatory Cytokine Production

**Figure 6:**
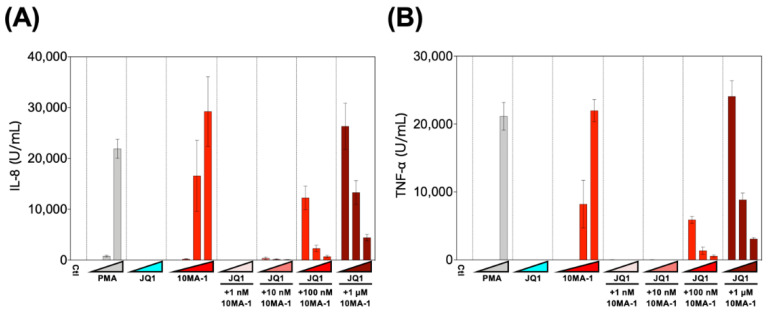
Pro-inflammatory cytokine production induced by the combined treatment of LRAs. PBMCs were treated with increasing concentrations of PMA (0.1 nM, 1 nM, or 10 nM), JQ1 (10 nM, 100 nM, or 1 µM), 10MA-1 (1 nM, 10 nM, 100 nM, or 1 µM), or JQ1 (10 nM, 100 nM, or 1 µM) and 10MA-1 (1 nM, 10 nM, 100 nM, or 1 µM) for 24 h. IL-8 (**A**) and TNF-α (**B**) in the culture supernatants were measured by ELISA. Experiments were performed in triplicate; the averages and standard deviations of the results obtained from the treated PBMCs are shown.

Finally, we examined the effect of the combined treatment of 10MA-1 with JQ1 on the induction of pro-inflammatory cytokines. PBMCs were treated with 1 nM, 10 nM, 100 nM, or 1 µM 10MA-1 with or without 10 nM, 100 nM, or 1 µM JQ1, respectively, for 24 h, and the amount of pro-inflammatory cytokines in the culture supernatants was evaluated (Figure 6). It was found that 10MA-1 induced IL-8 and TNF-α production in a dose-dependent manner, while JQ1 did not. When 10MA-1 was treated along with JQ1, the production of both cytokines in the supernatants decreased dose-dependently of JQ1, even at the highest concentration (1 µM) of 10MA-1. Notably, the amount of IL-6 and IL-1β produced in PBMCs by treating 10MA-1 with or without JQ1 was under the detection limit (data not shown). Consistent results were observed among PBMCs from three macaques (Figure S5). These results indicate that JQ1 successfully suppressed pro-inflammatory cytokine production due to global T cell activation by higher concentrations of 10MA-1 treatment.

## 4. Discussion

**Figure 7:**
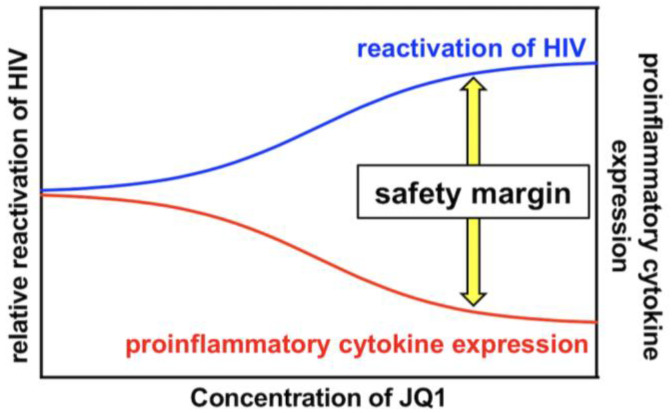
A kinetic model for the safety margin in the combined treatment of 10MA-1 with JQ1. The combined treatment of 10MA-1 with JQ1 leads to both synergistic HIV reactivation and reduced pro-inflammatory cytokine expression. As demonstrated by the relative difference in their activities of combined treatment, the safety margin is ameliorated dose-dependently of JQ1, whereas 10MA-1 alone merely exhibits the safety margin, irrespective of the dose of 10MA-1.

PKC activation induces the phosphorylation of IκB, eventually leading to NF-κB activation, which efficiently reactivates latently infected HIV [17,18]. Therefore, PKC activators have been considered robust LRAs [11,19,20]. However, PKC activators also developed various adverse events in previous clinical trials aiming at evaluating their anticancer efficacy [21,22,23,24], which hampered the intervention trial of PKC activators as LRAs [21]. A number of PKC activators, such as bryostatin-1, prostratin, PEP005, Ing-B, and Benzolactam compounds in combination with a BET inhibitor synergistically reactivated HIV [4,13,28,38,39]. Moreover, it was demonstrated that the combination treatment reduced global T cell activation and pro-inflammatory cytokine expression [28,29,30]. These results suggest that the combined use of a PKC activator and a BET inhibitor may overcome the safety issue of PKC activators as LRAs. However, whether a specific combination of a PKC activator with a BET inhibitor at optimized concentrations could achieve efficient HIV reactivation together with lowered and tolerable side reactions remains to be clarified. In this study, we showed that the combined treatment of 10MA-1 with JQ1 strongly and synergistically reactivated latently infected HIV (Figure 2 and Figure 3). The synergistic effect by the combined treatment of 10MA-1 with JQ1 was generally comparable with those by the combined treatment of bryostatin-1 with JQ1 or other BET inhibitors [28,29,30,38,39]. Further, JQ1 efficiently suppressed 10MA-1-induced global T cell activation as defined by CD25 expression and pro-inflammatory cytokine production (Figure 5 and Figure 6). Notably, JQ1 activity was efficient at higher concentrations of 10MA-1 up to 10 µM, suggesting that the safety margin of 10MA-1 may be improved by JQ1 in a dose-dependent manner (Figure 7). If it is the case in vivo, then the combined treatment of 10MA-1 with JQ1 would be a promising pair of LRAs for the future clinical study of “shock and kill” therapy.

It is reasonable to assume that the possible molecular mechanisms mediating the synergy between 10MA-1 and JQ1 may be comparable to those of combined treatment of bryostatin-1 and JQ1, as previously described; (i) bryostatin-1 activates NF-kB through the PKC pathway, which induces Tat protein expression as well as global T cell activation and pro-inflammatory cytokine production, (ii) Tat protein recruits p-TEFb released from BRD4 in the presence of JQ1 to the TAR element of HIV LTR, leading to HIV reactivation, and (iii) JQ1 competitively interacts with BRD4 and blocks p-TEFb recruitment, which inactivates phosphorylation of RNA polymerase II and transcription of pro-inflammatory cytokine genes [4,13,28,40]. Further analyses will be required to define the exact mechanism of 10MA-1 on PKC activation, by which the two distinct phenotypes are induced.

JQ1 showed substantial cytotoxicity in M8166 at higher concentrations; however, the combined treatment of JQ1 with 10MA-1 circumvented the cytotoxicity in both Jurkat and M8166 cells (Figure 4). This suggests that the combination may be appropriate in view of not only synergistic HIV reactivation but also lowered global T cell activation and cytotoxicity. JQ1 was tolerable in in vivo studies; oral administration of 10 mg/kg JQ1 or intraperitoneal administration of 50 mg/kg JQ1 did not show severe toxicity in mice [41]. Since LRAs should act transiently to reactivate latently infected HIV and JQ1 exerts a short half-life in vivo [42,43], it might establish the optimized protocol for the combined treatment of 10MA-1 with JQ1 to synergistically and sufficiently reactivate HIV and diminish the unfavorable side reactions in vivo.

In this study, we employed PBMCs from cynomolgus macaques, considering that we are planning to use a macaque model for the preclinical trial of 10MA-1 in combination with JQ1. As the cellular and molecular functions of macaques are comparable to those of humans, it is reasonable to consider that the results of the macaque T cell responses following treatment with LRAs may be applicable in humans. It will be necessary to confirm the present results of the combination LRAs in human PBMCs for clinical studies in the future.

10MA-1 is an analog of ATX as a PKC activator purified from a sea hare (*Stylocheilus longicauda*) [37]. Initially, the ability of ATX as an antitumor agent was shown [44]. After that, aplog-1 was synthesized as a simple and less lipophilic analog of ATX with antiproliferative and antitumor-promoting activities [45]. Kikumori et al. further synthesized 10MA-1, which bound to novel PKCs (δ, ε, η, and θ) approximately 10–20 times stronger than aplog-1, by installing a methyl group at position 10 of spiroketal moiety of aplog-1 (Figure 1) [31]. Studies show that 10MA-1 can be synthesized by 23 steps with standard reactions [33], which is fewer than bryostatin 1 or its potent analogs [46]. Developing a more concise synthetic route of 10MA-1 and its analogs is in progress in Irie’s laboratory, making in vivo study more feasible.

## Data Availability

The data presented in this study are available in article or Supplementary Material.

## Supplementary Materials

The following are available online at https://www.mdpi.com/article/10.3390/v13102037/s1, Figure S1: Kinetics of HIV reactivation in Jurkat 1G5 treated with LRAs as shown by RLU, Figure S2: The effect of lower concentrations of JQ1 on synergistic HIV reactivation by the combined treatment of LRAs, Figure S3: T cell activation induced by lower concentration of LRA combinations, Figure S4: T cell activation induced by a higher concentration of LRA combinations, Figure S5: Pro-inflammatory cytokine expression induced by the combined treatment of LRAs.
