# Supplementary material for: The Novel PKC Activator 10-Methyl-Aplog-1 Combined with JQ1 Induced Strong and Synergistic HIV Reactivation with Tolerable Global T Cell Activation
Source: Viruses. 2021 Oct 9;13(10):2037. doi: 10.3390/v13102037 (PMC8541327; doi:10.3390/v13102037)
Supplement: Supplementary file 1 [file viruses-13-02037-s001.zip › viruses-1368446-supplementary.pdf]

viruses

Supplementary Materials

The Novel PKC Activator 10-Methyl-Aplog-1 Combined with JQ1 Induced Strong and Synergistic HIV Reactivation with Tolerable Global T Cell Activation

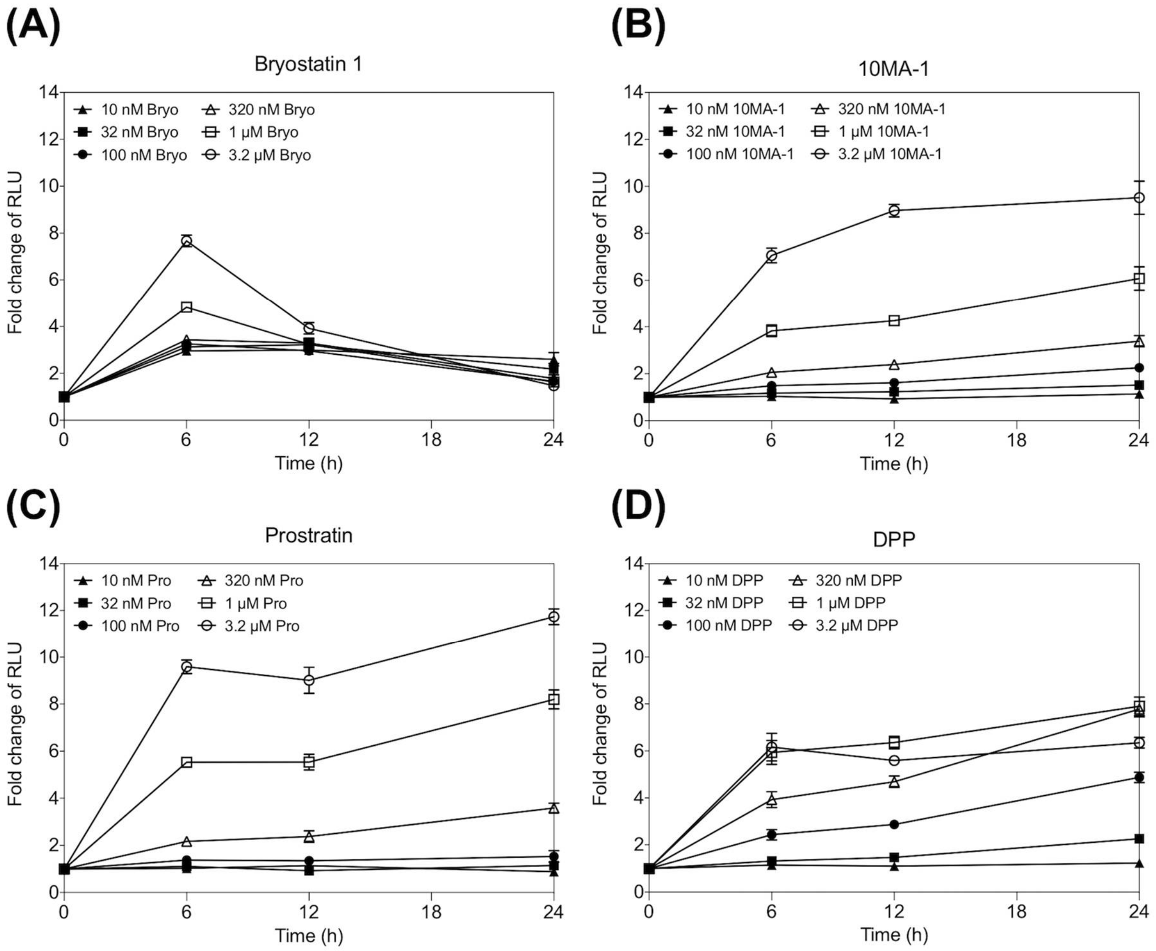

Figure S1. Kinetics of HIV reactivation in Jurkat 1G5 treated with LRAs as shown by RLU. Jurkat 1G5 was treated with (**A**) bryostatin 1, (**B**) 10MA-1, (**C**) prostratin, or (**D**) DPP at the concentration of 10 nM, 32 nM, 100nM, 320 nM, 1 μM, or 3.2 μM, respectively. At 0, 6, 12, and 24 h after LRA treatment, a fold increase of RLU in the treated cells compared with untreated cells was measured. We performed experiments in triplicate; averages and standard deviations of the results were shown.

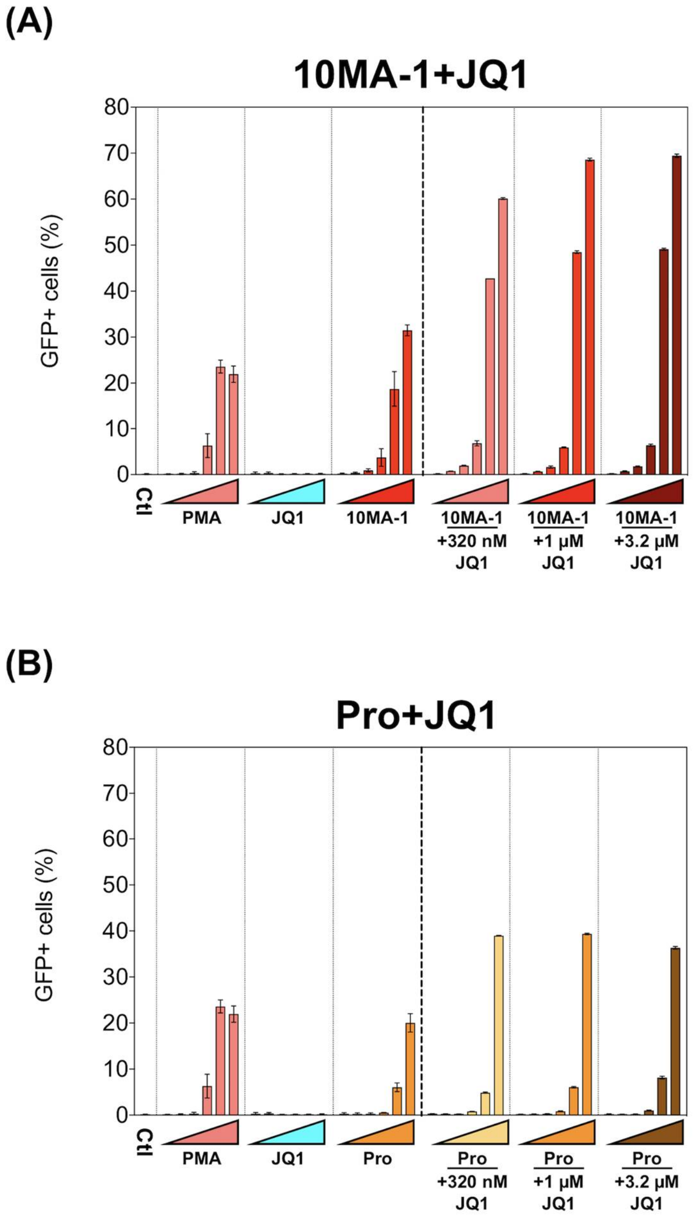

Figure S2. The effect of lower concentrations of JQ1 on synergistic HIV reactivation by the combined treatment of LRAs. J-Lat 9.2 was treated with 10MA-1 (**A**) or prostratin (**B**) with or without JQ1 for 48 h, then the percentage of cells expressing GFP was analyzed by flow cytometry. Experiments were performed in triplicate; averages and standard deviations of the results were shown. The LRAs were used in these assays at a concentration of 10 nM, 32 nM, 100 nM, 320 nM, 1 μM, or 3.2 μM for each LRA or along with 320 nM, 1 μM, or 3.2 μM of JQ1, respectively. PMA was used as a positive control at a concentration of 0.1 nM, 0.32 nM, 1 nM, 3.2 nM, 10 nM, or 32 nM.

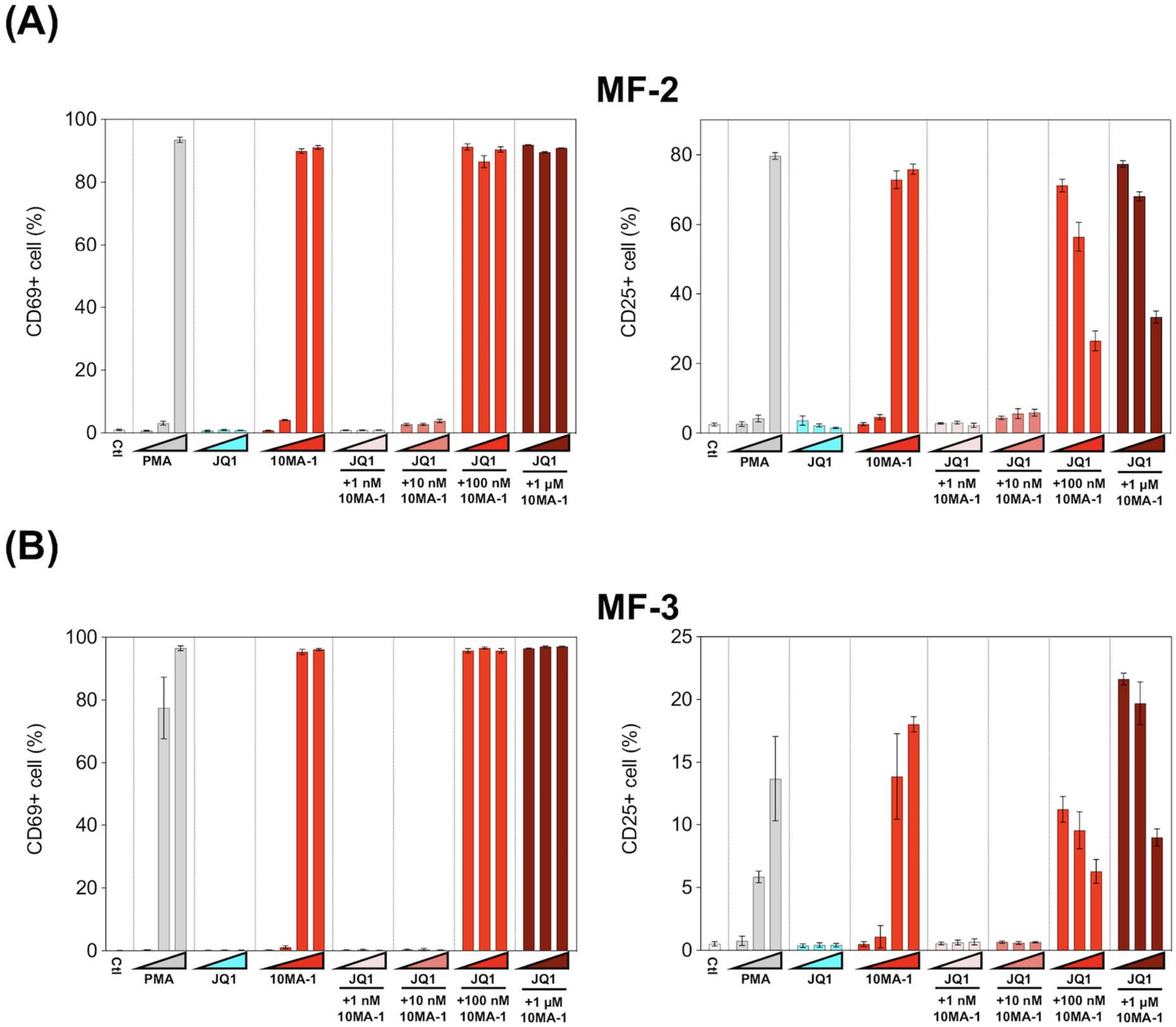

Figure S3. T cell activation induced by lower concentration of LRA combinations**.** PBMCs were untreated or treated with increasing concentrations of PMA (0.1 nM, 1 nM or 10 nM), JQ1 (10 nM, 100 nM, or 1 μM), 10MA-1 (1 nM, 10 nM, 100 nM, or 1 μM), or JQ1 (10 nM, 100 nM, or 1 μM) and 10MA-1 (1 nM, 10 nM, 100 nM, or 1 μM), respectively, for 24 hrs. The CD4+ T lymphocytes were measured for the positivity of CD69 (left) and CD25 (right) by flow cytometry. We performed experiments in triplicate; the averages and standard deviations of the results obtained from the PBMCs of the other two macaques than that shown in Figure 5A were shown (A: MF-2, B: MF-3).

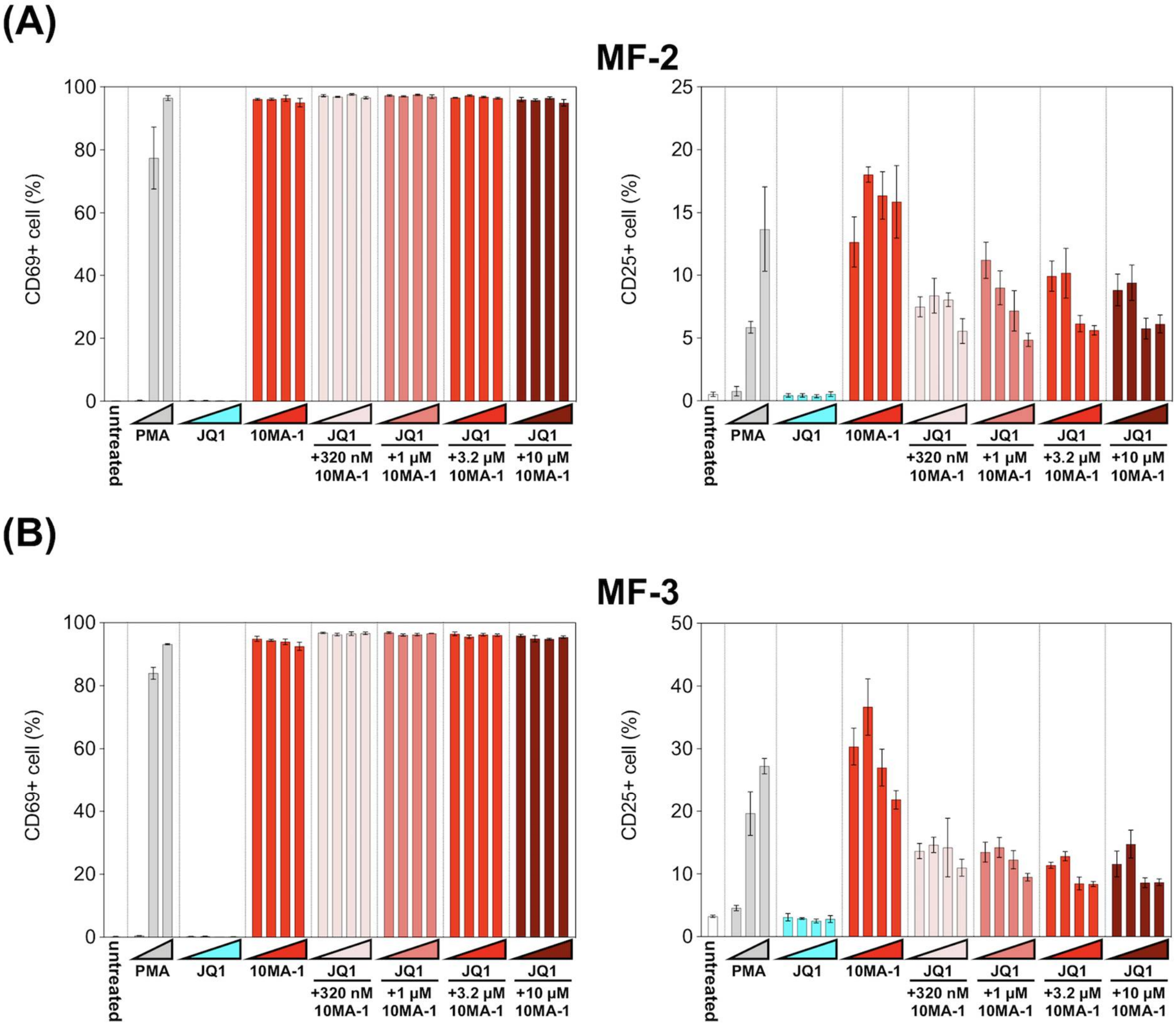

Figure S4. T cell activation induced by a higher concentration of LRA combinations. PBMCs were untreated or treated with the increasing concentration of PMA (0.1 nM, 1 nM, or 10 nM), JQ1 (320 nM, 1 μM, 3.2 μM, or 10 μM), 10MA-1 (320 nM, 1 μM, 3.2 μM, or 10 μM), or JQ1 (320 nM, 1 μM, 3.2 μM, or 10 μM) and 10MA-1 (320 nM, 1 μM, 3.2 μM, or 10 μM) for 24 h. The CD4+ T lymphocytes were examined for the positivity of CD69 (left) and CD25 (right) by flow cytometry. We performed experiments in triplicate; the averages and standard deviations of the results obtained from the PBMCs of the other two macaques than that shown in Figure 5A were shown (A: MF-2, B: MF-3).

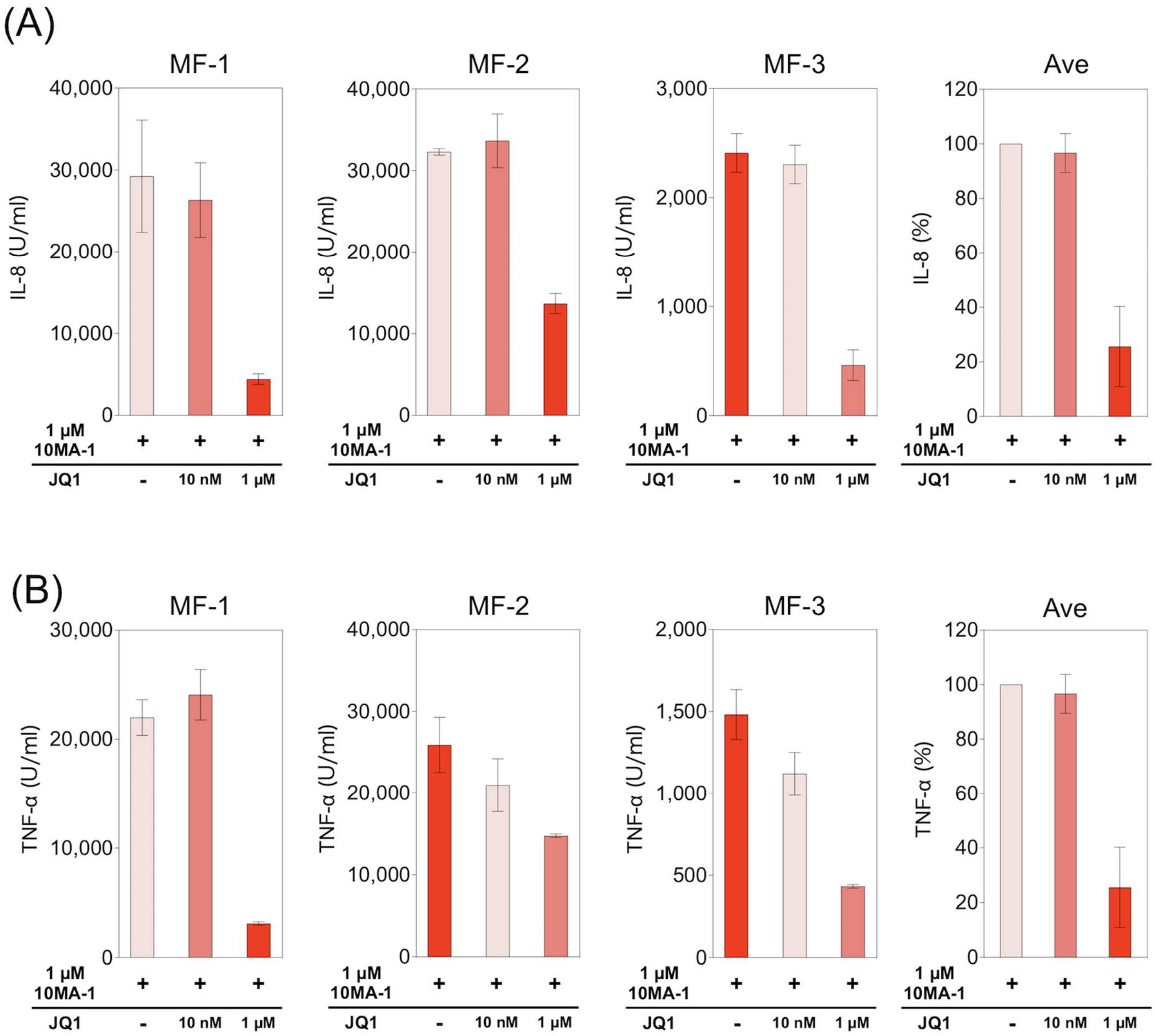

Figure S5. Pro-inflammatory cytokine expression induced by the combined treatment of LRAs**.** PBMCs from three macaques (MF-1, 2, 3) were treated with 1 μM 10MA-1 with or without JQ1 (10 nM or 1 μM) for 24 h. IL-8 (**A**) and TNF-*α* (**B**) in the culture supernatants were measured by ELISA. We performed experiments in triplicate; the averages and standard deviations of the results were shown. Ave indicates the averages of IL-8 and TNF-*α* expression in PBMCs from three monkeys after LRA treatment was calculated, as the amount of IL-8 and TNF-*α* by 1 μM 10MA-1 treatment was set to 100%.
